# Response of microbial communities and physical and chemical properties in inoculated soils with *Fusarium oxysporum* Schl. F. sp. *Benincasae* to different root exudates resistant to fusarium wilt

**DOI:** 10.3389/fmicb.2025.1595426

**Published:** 2025-07-17

**Authors:** Haolong Wu, Junyu Fu, Daolong Liao, Yilin Chen, Zhen Zhao, Zifan Liu, Bin Zhou

**Affiliations:** ^1^School of Tropical Agriculture and Forestry, Hainan University, Danzhou, China; ^2^Institute of Vegetables, Hainan Academy of Agricultural Sciences, Danzhou, China; ^3^Tropical Crops Genetic Resources Institute, Chinese Academy of Tropical Agricultural Sciences, Danzhou, China

**Keywords:** inoculated soils, wax gourd wilt, root exudates, metabolome, microbial diversity

## Abstract

Wax gourd wilt (WGW) is a destructive soil-borne disease, and grafting pumpkin rootstocks offers effective and eco-friendly control. “Haizhan 1” pumpkin rootstock exhibits superior disease resistance compared to *Cucurbita ficifolia*, with root exudates improving soil microecology. However, the specific root exudate components involved in wilt resistance and their effects on soil microbial diversity remain unclear. In this paper, “Haizhan 1” pumpkin, *Cucurbita ficifolia* pumpkin and “Tiezhu 168” wax gourd are taken as rootstock materials to graft and obtain the five kinds of experimental subjects (H_T, B_T, H_H, B_B, T_T). Root exudates from five grafted combinations were analyzed by using metabolomics. Ten key metabolites that have direct fungicidal effects and indirect regulatory effects on disease resistance were identified, including Melilotoside A, a substance unique to pumpkin-wax gourd grafting. Potted experiments revealed that root exudates from resistant (H_T), moderately resistant (B_T), and susceptible (T_T) plants altered soil microbial communities under *Fusarium oxysporum* challenge. High-throughput sequencing identified six key bacteria linked to disease resistance: *norank_f__norank_o_0319-6G20*, *Haliangium*, *norank_f__norank_o__norank_c__OLB14*, *Thermomonas*, *Brevundimonas*, and *Gemmatimonas*. Correlation analysis highlighted the interaction between root exudate metabolites and soil microbes. This study clarifies the role of root exudates in WGW resistance and provides a foundation for developing biocontrol strategies.

## 1 Introduction

Wax gourd wilt, caused by the *Fusarium oxysporum* Schl. F. sp. *Benincasae* (FOB), is one of the devastating soil-borne diseases. The disease persists throughout the crop’s growth cycle, causing whole-plant death or severe yield reduction, ultimately resulting in substantial economic losses. Current control strategies include developing resistant varieties, applying chemical fungicides, and utilizing biological control agents (Shi et al., 2022). However, breeding resistant varieties is time-consuming (Ao et al., 2021), and cultivar instability often hinders rapid field deployment (Wang et al., 2019b). Overuse of chemical fungicides harms the environment and is unsustainable (Cai et al., 2015). Although biological control avoids environmental pollution and pesticide residues, challenges remain, such as strain stability, rapid degradation, and high biopesticide costs (Rector, 2009). Therefore, a more environmentally friendly and effective approach is required for wax gourd wilt control.

Grafting cultivation effectively prevents and controls wax gourd wilt, offering operational simplicity, low cost, yield stability, and rapid field applicability (Chen and Wu, 2021; Evans et al., 2022). Rootstock selection determines grafting-induced wilt resistance. From 12 tested germplasms, Zeng identified three wax gourd rootstocks exhibiting high wilt resistance (Zeng, 2021). Traditional *Cucurbita ficifolia* rootstock (B_T) shows moderate resistance (wilt index 42.6–52.6), whereas ‘Haizhan 1’ confers complete immunity (index 0) (Zhou, 2017; Zhu et al., 2018).

Plant disease resistance correlates with root exudate profiles (Buxton, 1962). Root exudates mediate plant–soil communication and regulate plant-pathogen interactions. Soil microorganisms show selective responses to root exudates components, shaping distinct microbial communities (Tan et al., 2020). Specifically, aromatic acids reduce overall microbial community diversity, while amino acids and nitrogenous compounds selectively decrease bacterial community diversity (Zhalnina et al., 2018; Gu et al., 2020). By reshaping rhizosphere microbiomes, root exudates modulate soil-borne disease incidence (Hu et al., 2018).

Grafting physically joins two genetically distinct plants: a rootstock and a scion. Rootstock-scion interactions alter root exudate composition (Zhang et al., 2020). Previous studies revealed that grafted watermelon roots secrete more diverse proteins than self-rooted plants or rootstocks alone (Ling et al., 2013); Rootstock selection influences amino acid profiles in eggplant root exudates (Zhou et al., 2001). Our team demonstrated that H_T rootstocks confer wilt resistance through physical barrier formation (Fu et al., 2024); and H_T root exudates improve soil quality and sustain beneficial micro-ecosystems more effectively than self-grafted wax gourd exudates (Fu et al., 2022). However, the functional root exudate components responsible for Fusarium wilt suppression and their mechanisms of reshaping soil microbial communities remain unclear. We hypothesize that resistant rootstocks (e.g., “Haizhan 1”) release distinct metabolites that either directly inhibit Fusarium oxysporum or indirectly recruit beneficial microbes. In this paper, metabolomics and high-throughput sequencing were combined to (1) identify key functional components in root exudates and (2) characterize their associated microbial taxa, aiming to elucidate the rhizosphere mechanisms underlying grafting-induced resistance. These findings establish a foundation for developing biocontrol agents against wax gourd wilt.

## 2 Materials and methods

### 2.1 Experimental materials

The *Fusarium oxysporum* Schl. F. sp. *benincasae* physiological strain No. 1 (denoted as FOB) was provided by the Vegetable Research Institute of Guangxi Academy of Agricultural Sciences.

The experimental soil was collected from Luofu Village, Rui Xi Town, Chengmai County, Hainan Province, and its basic physicochemical properties were as follows: pH 5.94, organic matter 0.51%, alkaline-hydrolyzed nitrogen 28.1 mg/kg, available phosphorus 103.3 mg/kg and available potassium 129.6 mg/kg. The soil was sieved to remove debris, air-dried, passed through an 80-mesh sieve, and stored.

The varieties of wax gourd were “Tiezhu 168” (provided by Guangdong Kenong Vegetable Seed Co., Ltd.). The pumpkin varieties were “Haizhan 1” (provided by Vegetable Research Institute of Hainan Academy of Agricultural Sciences) and *Cucurbita ficifolia* (provided by Shouguang Jinguo Agricultural Technology Co., Ltd.). Five types of seedlings were obtained by referring to the seedling raising method of Fu et al.: susceptible self-grafted wax gourd (T_T); medium resistant self-grafted Haizhan (H_H), grafted *Cucurbita ficifolia* (B_T) and self-grafted *Cucurbita ficifolia* (B_B); immune plant grafted Haizhan (H_T) (Fu et al., 2024).

### 2.2 Collection and concentration of root exudates

#### 2.2.1 Collection of root exudates

Seedlings were transferred to the laboratory at the Haidian campus, Hainan University when the plants had grown two leaves, and the root exudates were collected by the mixed soil-hydroponic method (Liu, 2022). In brief, the seedlings were removed from the experimental soil, and their roots were thoroughly washed, and transferred to sterile water for the secondary cultivation. Each seedling was placed in a brown test tube and sterile water was added until the roots were fully submerged. Seedlings were incubated in a light incubator, where the alternating temperature of day and night (12 h/12 h) was 26°C/21°C and the light intensity was 2000 lx. Root exudates were collected once daily at 24 h, 48 h, and 72 h. The collected solution represented the crude extract of root exudates, which was stored at 4°C. After the root exudates were collected for the last time, the roots of the five seedling types were rinsed with distilled water, blotted dry with filter paper, and weighed to determine fresh weight.

#### 2.2.2 Concentration of root exudates

The collected crude extracts of root exudates were filtered through a 0.22 μm aqueous membrane using respective vacuum filtration devices (Liu, 2022). Each 50 mL aliquot of root exudates was passed through a solid phase extraction cartridge containing 2 mL of XAD-4 resin. The solution was cycled through the cartridge three times to ensure complete adsorption of root exudates onto the resin. Then, the cartridge was eluted with methanol (50 mL), and finally methanol was evaporated from the eluate to complete dryness by vacuum rotary evaporator under reduced pressure at 40°C. The resulting residue was dissolved in 15 mL of deionized water to obtain a concentrated solution of root exudates (Xu et al., 2012).

### 2.3 Preparation of spore suspension and inoculated soils

Firstly, the *Fusarium oxysporum* spore suspension with a concentration of 2.75 × 10^7^ CFU/g was prepared, and the experimental diluent was obtained by diluting the spore suspension with sterile water at a ratio of 1:4 (v/v). The experimental diluent was poured into the sifted soil at a ratio of soil: bacterial solution = 6 g: 1 mL (w/v), and mixed thoroughly to obtain the inoculated soil (Fu et al., 2024).

### 2.4 Experimental design

To simulate root exudation in the rhizosphere, the inoculated soil (120 g) was placed in 100 mL beakers. Three root exudates treatments (T_T, H_T, and B_T,) were established with four replicates each. Root exudates were prepared at a concentration of 0.05 g/mL (equivalent to 0.05 g of exudates per mL sterile water, based on fresh root weight). The root exudates were added into a beaker filled with inoculated soils and cultivated in a constant temperature incubator at 28°C. The treatment procedure consisted of adding 7 mL of root exudates to the inoculated soil on the first day after inoculation, followed by subsequent additions at 3-day intervals (Afzal et al., 2024). All exudates were applied to a fixed point at the center of each beaker to mimic natural root secretion.

### 2.5 Determination indexes and methods

#### 2.5.1 Determination of soil physical and chemical properties and enzyme activity

Sampling method: On the 20th day after treatment, soil sample were collected from the center of each beaker using a 30-mm-diameter corer. Samples were naturally air-dried at room temperature for subsequent analysis. Soil physicochemical properties including pH, organic matter (OM), alkaline-hydrolyzed nitrogen (Alkali-N), available phosphorus (AP), available potassium (AK), total nitrogen (TN), total phosphorus (TP) and total potassium (TK) were determined according to standard methods (Liu et al., 2023).

Soil enzyme activities were measured using commercial kits (Beijing Solarbio Technology Co., Ltd.) following the manufacturer’s protocols: urease (S-UE), sucrase (S-SC), catalase (S-CAT), and acid phosphatase (S-ACP) (Hou et al., 2020).

#### 2.5.2 Soil DNA extraction and Illumina MiSeq DNA sequencing

Soil DNA extraction and sequencing were performed as follows: soil samples collected as described in section 2.5.1 were immediately frozen in liquid nitrogen and stored at −80°C until processing. Total genomic DNA of the microbial community was extracted using the E. Z. N. A.^®^ soil DNA kit (Omega Bio-tek, Norcross, GA, United States). DNA quality was verified by 1% agarose gel electrophoresis, and concentration and purity was determined using a NanoDrop2000 spectrophotometer (Thermo Fisher Scientific, Waltham, MA, United States).

For bacterial community analysis, the V3-V4 region of 16S rRNA genes was amplified using barcoded primers 338F (5′-ACTCCTACGGGAGGCAGCAG-3′) and 806R (5′-GACTACHVGGGTWTCTAAT-3′). Fungal ITS1-ITS2 regions were amplified with primers ITS1F (5′-CTGGTCATTTAGGAGAGATA-3′) and ITS2R (5′-GCTGTGTTTACTGATGC-3′). The procedure of PCR amplification was as follows: initial denaturation at 95°C for 3 min, followed by 27 cycles of denaturing at 95°C for 30 s, annealing at 55°C for 30 s and extension at 72°C for 45 s, and single extension at 72°C for 10 min, and end used at 10°C. The PCR product was extracted from 2% agarose gel and purified using the PCR Clean-Up Kit (YuHua, Shanghai, China) according to manufacturer’s instructions and quantified using Qubit 4.0 (Thermo Fisher Scientific, Waltham, MA, United States). The preparation of the amplicon library and Illumina MiSeq double ended DNA sequencing were carried out at Majorbio Bio-Pharm Technology Co. Ltd. (Shanghai, China). Raw data for high-throughput sequencing were deposited in the National Center for Biotechnology Information.^1^

### 2.6 Metabolome analysis of root exudates

The collection and concentration of root exudates followed the procedure described in section 2.1. The five collected types of concentrated root exudates (T_T, H_T, B_T, H_H and B_B) were freeze-dried to a powder form. For metabolite extraction, 10 ± 5 mg of freeze-dried powder was accurately weighed into a 2 mL centrifuge tube, mixed with 160 μL of extraction solution (methanol: water = 4:1, v/v), and subjected to low-temperature ultrasonic extraction for 30 min (40 kHz, 5°C). The mixture was centrifuged for 15 min (13,000 g, 4°C) and transfer the supernatant was transferred to an injection vial with an inner tube for LC–MS analysis.

Quality control (QC) samples were prepared by mixing equal volumes of extraction solutions from all samples. Each QC sample was processed identically to the experimental samples. During the instrument analysis, a QC sample is inserted to each 5–15 analytical samples to monitor system stability. Metabolite profiling was performed using an ultra-high performance liquid chromatography-tandem Fourier transform mass spectrometry (UHPLC-Q Exactive) system equipped with an ACQUITY UPLCBEH C18 chromatographic column (100 mm × 2.1 mm i.d., 1.7 μm; Waters, Milford, MA, United States). The raw data were processed using Progenes QI v3.0 software (Waters Corporation, Milford, United States) for baseline filtering, peak identification, integration, retention time correction, and peak alignment. Afterwards, the software was used for feature peak search library identification, matching MS and MS/MS mass spectrometry information with plant specific metabolite database (MJDBPM) built by Majorbio. The max qualitative error of MS was set to be 10 ppm, and metabolites were identified based on the secondary mass spectrometry matching score. Differential metabolites were screened using the following criteria: (1) fold change > 2 or < 0.5 (log₂FC > 1 or < −1), and (2) *p*-value < 0.05 from Student’s *t*-test.

### 2.7 Statistical analysis

The soil physicochemical properties and enzyme activity results were expressed as mean ± standard error. DPS 9.01 software was used to perform statistical analysis on the data through a completely random design one-way analysis of variance. Origin 2021 software was used for correlation heatmap analysis. All data analysis for high-throughput sequencing and metabolomics testing was conducted on the Majorbio Cloud Platform.^2^

The analytical workflow for screening effective components to wilt disease resistance in root exudates was illustrated in Supplementary Figure S1, while the approach for identifying key microorganisms in soil inoculated with bacteria affected by root exudates was shown in Supplementary Figure S2.

## 3 Results

### 3.1 Different grafting methods altered the composition of root exudates

**Figure 1:**
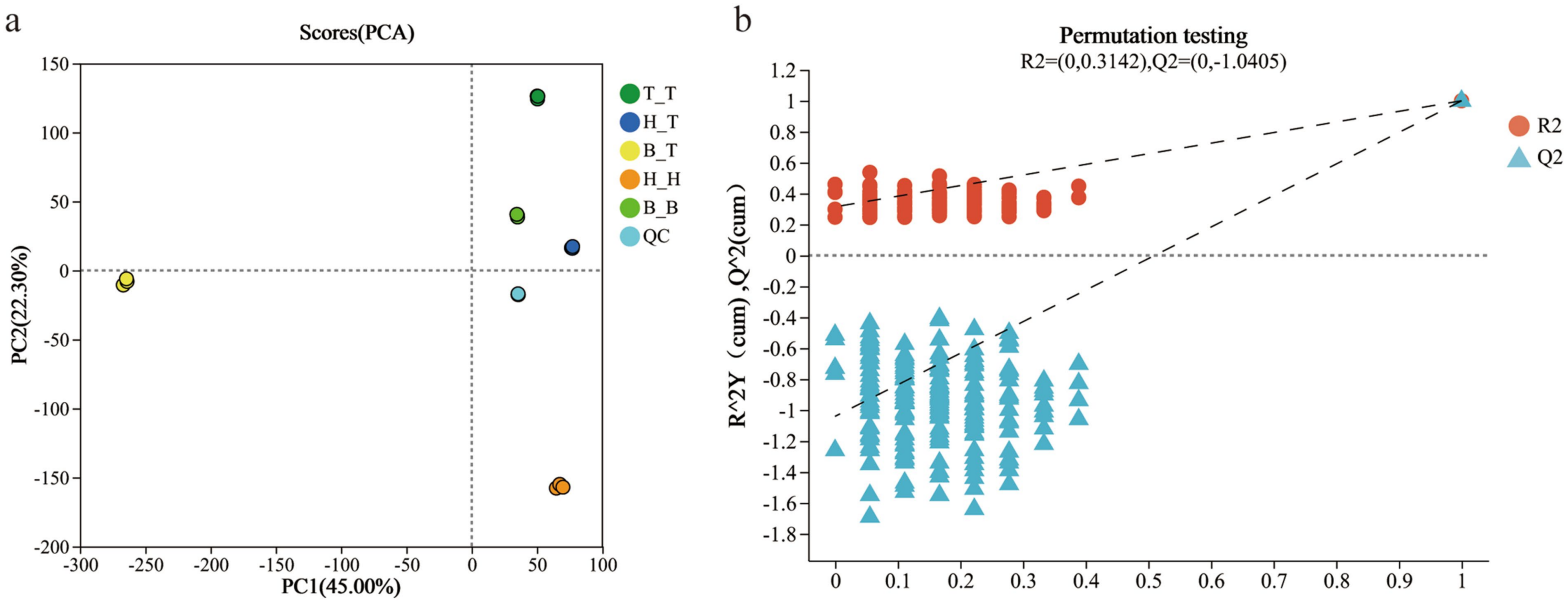
Metabolome principal component analysis **(a)** and model verification results **(b)** of different root exudates.

The good grouping of QC samples indicates that the biological analysis and data quality are good. The three times repetitions of the five types of root exudates treatments are concentrated or even overlapping in the distribution in the analysis figures indicating that the differences within each treatment are slight. There are significant differences between the samples of groups H_T, T_T, H_H, B_T, and B_B and they are far apart from each other, which are within the 95% confidence interval. The differences in metabolites between the samples are significant (Figure 1a).

The verification results of the PLS-DA model showed that R^2^ was all above Q^2^, and the intercept of the regression line of Q^2^ and the vertical axis of Y was −1.0405 (<0.05), indicating that the model was well fitted and predictable, and suitable for subsequent data analysis (Figure 1b).

**Figure 2:**
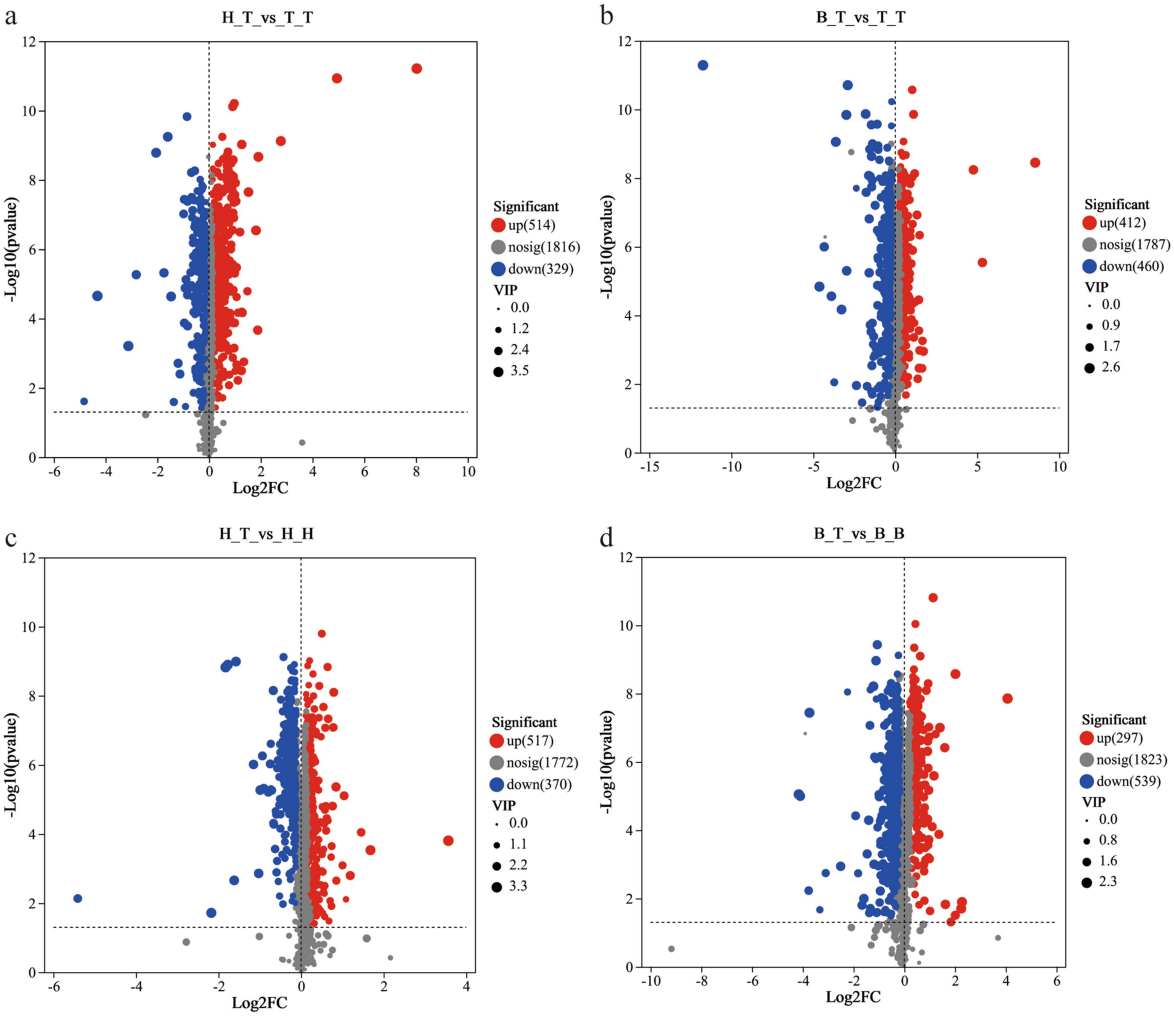
Volcano maps of different comparison groups **(a-d)**. The red dots represent significantly up-regulated metabolites, the blue dots represent significantly down-regulated metabolites, and the gray dots represent non-significantly differentiated metabolites.

The results showed that there were 843 significantly different metabolites (514 up-regulated and 329 down-regulated) in the comparison group between H_T and T_T; there were 872 significantly different metabolites (412 up-regulated and 460 down-regulated) in the comparison group between B_T and T_T; there were 887 significantly different metabolites (517 up-regulated and 370 down regulated) in the comparison group between H_T and H_H; there were 836 significantly different metabolites (297 up-regulated and 539 down-regulated) in the comparison group between B_T and B_B (Figure 2).

**Figure 3:**
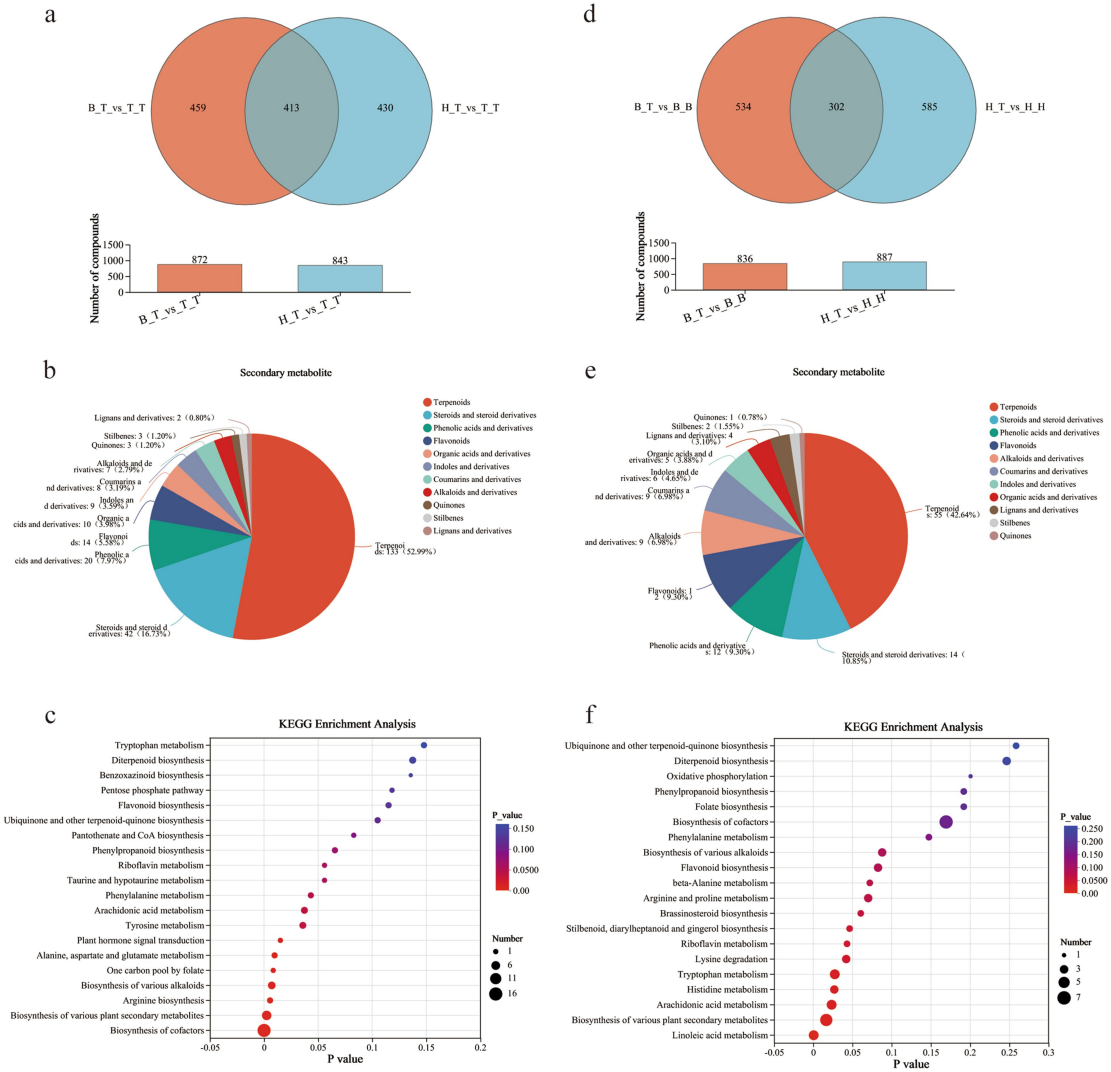
Venn diagram **(a,d)**, plant secondary classification of common metabolites **(b,e)**, and KEGG enrichment pathway diagram **(c,f)** of different comparison groups.

Venn diagram analysis was performed on the groups of self-grafted wax gourd (T_T) and grafted wax gourd (H_T and B_T), i.e., H_T vs. T_T and B_T vs. T_T, and 413 compounds with significant differences in common were found (Figure 3a). Among them, the main compounds ere terpenoids (52.99%), steroids and steroid derivatives (16.73%), phenolic acids and their derivatives (7.97%), etc. (Figure 3b). Ten significant enrichment pathways were shown in the KEGG enrichment pathway, revealed that the main compounds were involved in the biosynthesis of cofactors, various plant secondary metabolites, and arginine (Figure 3c).

Conduct A one-factor analysis of variance was conducted on the 413 significantly differential metabolites between the H_T vs. T_T and B_T vs. T_T comparison groups. The results revealed that the relative contents of 132 metabolites in the H_T and B_T groups were significantly higher than T_T, on this basis, a comparison was conducted between H_T and B_T, and the results revealed that the relative content of 56 metabolites in H_T was significantly higher than that in B_T. The above 56 metabolites may be key substances for the different resistance of grafted wax gourd to wilt disease, but the functions of these key metabolites need further clarification. Functional searches were conducted on the 56 metabolites (Supplementary Table S1), and it was identified that several metabolites with antibacterial and anti-inflammatory functions such as Ectoine, 4-Coumaroylputrescine, Desmethylxanthohumol, Curdione, Perillyl alcohol, Oryzalide B, Barringtogenol C, Melilotoside A, Cucurbitacin C, and Cucurbitacin D.

To clarify whether the differences in metabolite content are related to the rootstock-scion interaction effect, Venn diagram analysis and KEGG analysis were performed on grafted wax gourds with pumpkin rootstock and corresponding self-grafted pumpkins, i.e., H_T vs. H_H and B_T vs. B_B, and 302 common significant differential metabolites were found (Figure 3d). The main compounds were terpenes (42.64%), steroids and steroid derivatives (10.85%), phenolic acids and their derivatives (9.30%), etc. (Figure 3e). The KEGG enrichment pathway showed that eight enrichment pathways, including linoleic acid metabolism, biosynthesis of various plant secondary metabolites, and arachidonic acid metabolism, were significant in this shared compound (Figure 3f).

Single factor analysis of variance (one-way ANOVA) was conducted on 302 significantly different metabolites in the root exudates of grafted wax gourd compared to self-grafted pumpkin (H_T vs. H_H and B_T vs. B_B). It was found that the relative content of 106 metabolites in the root exudates of grafted wax gourd was significantly higher than that of self-grafted pumpkin (Supplementary Table S1).

Then, 56 differential metabolites were screened from the root exudates of plants with wax gourd as the scion, and 106 differential metabolites were screened from the root exudates of plants with pumpkin as the rootstock. It was found that the only common metabolite in the 2 groups was Melilotoside A (Supplementary Table S1). This identified that Melilotoside A is a metabolite generated by the interaction of pumpkin rootstock and wax gourd scion, which affects the disease resistance of grafted wax gourd.

### 3.2 Effects of root exudates on soil physical and chemical properties and enzyme activity

Compared with T_T (susceptible) root exudates, the pH, OM, Alkali-N, TN, and TK in the soil with H_T (immune) and B_T (medium resistance) root exudates are significantly increased. There are no significant differences in AP, AK, S-CAT, S-UE, and S-SC between the soil with H_T and B_T root exudates. The TP in the soil with H_T root exudates is significantly increased and the S-ACP is significantly decreased, while TP and S-ACP in the soil with B_T root exudates are not significantly different (Table 1). The above results indicate that compared to T_T, the addition H_T and B_T root exudates have a positive effect on most soil nutrients.

**Table 1:** Soil physical and chemical properties and enzyme activity.

	pH	OM	Alkali-N	AP	AK	TN	TP	TK	S-CAT	S-ACP	S-UE	S-SC
		g/kg	mg/kg	mg/kg	mg/kg	g/kg	g/kg	g/kg	U/g	U/mg	U/g	U/g
B_T	6.36 ± 0.01a	9.18 ± 0.08a	56.52 ± 0.87a	67.36 ± 0.13a	188.99 ± 3.74b	0.99 ± 0.01a	0.25 ± 0.00b	18.36 ± 0.05b	2.71 ± 0.36a	11.56 ± 0.49a	49.96 ± 3.64a	6.52 ± 0.63a
H_T	6.33 ± 0.01b	8.7 ± 0.05b	54.89 ± 1.08a	66.83 ± 0.14a	201.36 ± 3.36a	0.93 ± 0.01b	0.27 ± 0.00a	19.83 ± 0.40a	1.58 ± 0.29b	8.42 ± 0.89b	59.3 ± 2.60a	7.08 ± 0.08a
T_T	5.77 ± 0.00c	7.78 ± 0.05c	50.22 ± 1.00b	70.03 ± 1.86a	196.6 ± 1.16ab	0.72 ± 0.01c	0.25 ± 0.01b	16.88 ± 0.07c	1.9 ± 0.21ab	10.88 ± 0.31a	58.91 ± 6.49a	5.55 ± 1.06a

ANOVA, Duncan’s test; the letters indicate *p* < 0.05; Datas are means ± standard error of four replicates.

### 3.3 Effects of root exudates on diversity of soil microbial communities

**Figure 4:**
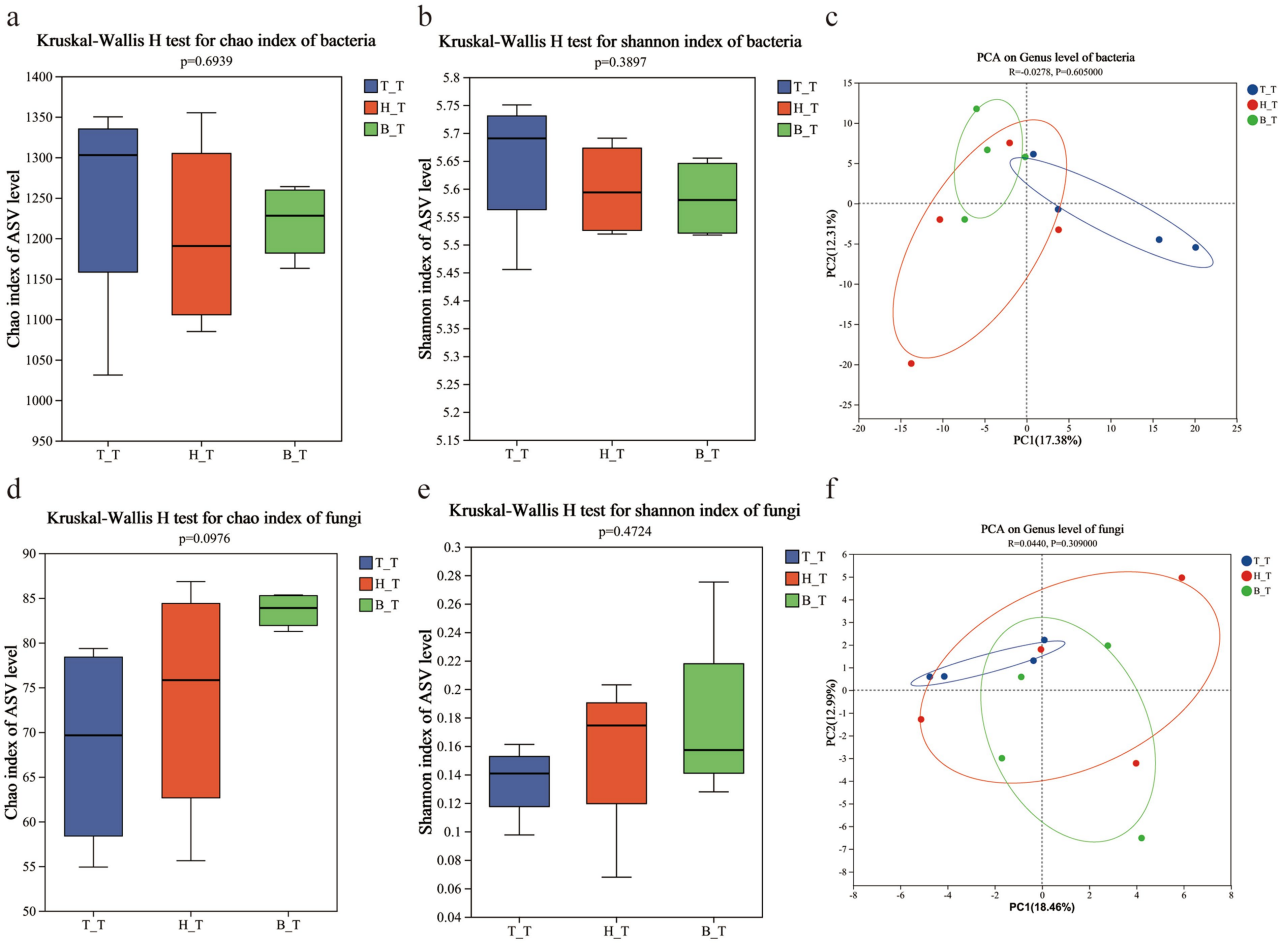
Alpha diversity (Chao and Shannon index) and PCA analysis of the communities of bacteria **(a–c)** and fungi **(d–f)** in inoculated soils with different root exudates added.

Perform diversity index difference test the soil microorganisms with three types of root exudates, respectively T_T, B_T, and H_T. The results revealed that soil under the addition of three root exudates, there is no significant difference in the bacterial chao index (*p* = 0.6939) (Figure 4a) and Shannon index (*p* = 0.3897) (Figure 4b); there is no significant difference in the fungal chao index (*p* = 0.0976) (Figure 4d) and Shannon index (*p* = 0.4724) (Figure 4e). These figures indicate that the α-diversity (Chao and Shannon indices) of bacteria and fungi in the soil is less affected by root exudates and the difference is not significant.

Based on PCA analysis, the soil microbial community structure with three types of root exudates T_T, B_T, and H_T, are studied to visualize the enrichment of core microorganisms in the process of the occurrence of wilt. The bacterial and fungal in the soils with three types of root exudates were partial aggregation (Figures 4c,f). The dispersion in PCA indicates that the composition of bacterial and fungal microbial communities in the soil is affected slightly on the addition of plants root exudates, and the difference is not significant.

### 3.4 Effects of root exudates on composition of soil microbial communities

**Figure 5:**
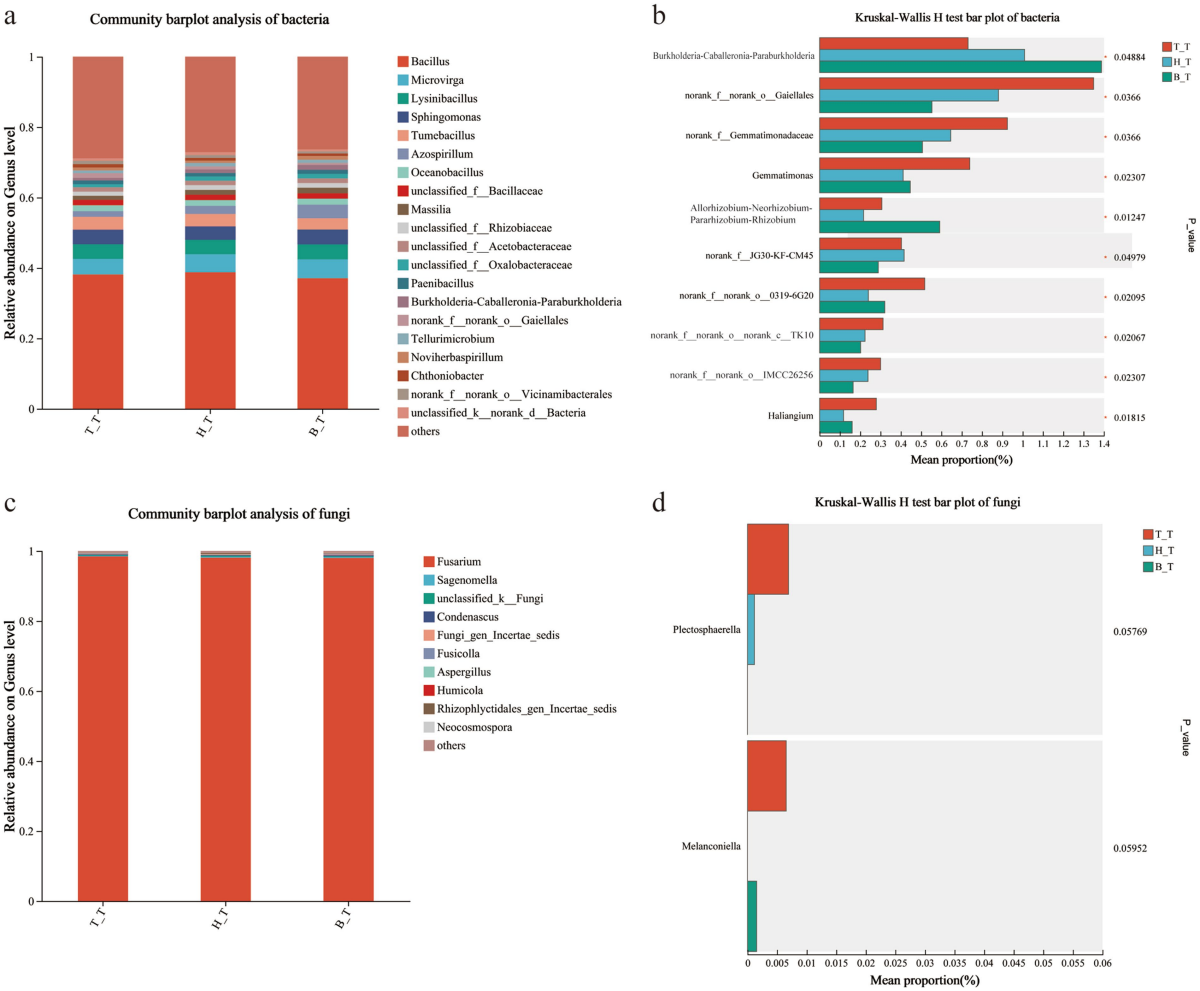
Bar diagram and multi-group comparison diagram of the communities of bacteria **(a,b)** and fungi **(c,d)** in inoculated soils with different root exudates added.

The community structure and composition of bacteria in the soil with three types of root exudates are similar, and the dominant bacteria in the soil are mainly *Bacillus*, *Tumebacillus*, *Microvirga*, *Lysinibacillus*, and *Sphingomonas*. Among them, the relative abundance of Bacillus was 37.09% ~ 38.80%, which was higher in the soil with H_T root exudates (Figure 5a). In the fungal community, *Fusarium*, *Sagenomella*, *Fusicolla*, *Condenascus*, and *Fungi_gen_incertae_sedis* are the main dominant genera. Among them, the relative abundance of Fusarium was 98.03% ~ 98.48%, with the highest abundance in the soil with T_T root exudates (Figure 5b).

Taking *p* < 0.05 as the standard, compare the soil microorganisms with three types of root exudates added, and screen out the top 20 species with the average sum of abundance. Among them, *Thermincola* and *Brevundimonas* showed significant differences in relative abundance, with H_T > B_T > T_T; The relative abundance showed a pattern of H_T < B_T < T_T, and the bacteria with significant differences were *norank_f__norank_o0319-6G20*, *Gemmatimonas*, *Haliangium*, *Thermomonas*, and *norank_f__norank_o__norank_c__OLB14* (Figure 5c); However, there is no significant difference in soil fungi (Figure 5d).

### 3.5 Correlation between microbial communities, soil physical and chemical properties, and enzyme activities

**Figure 6:**
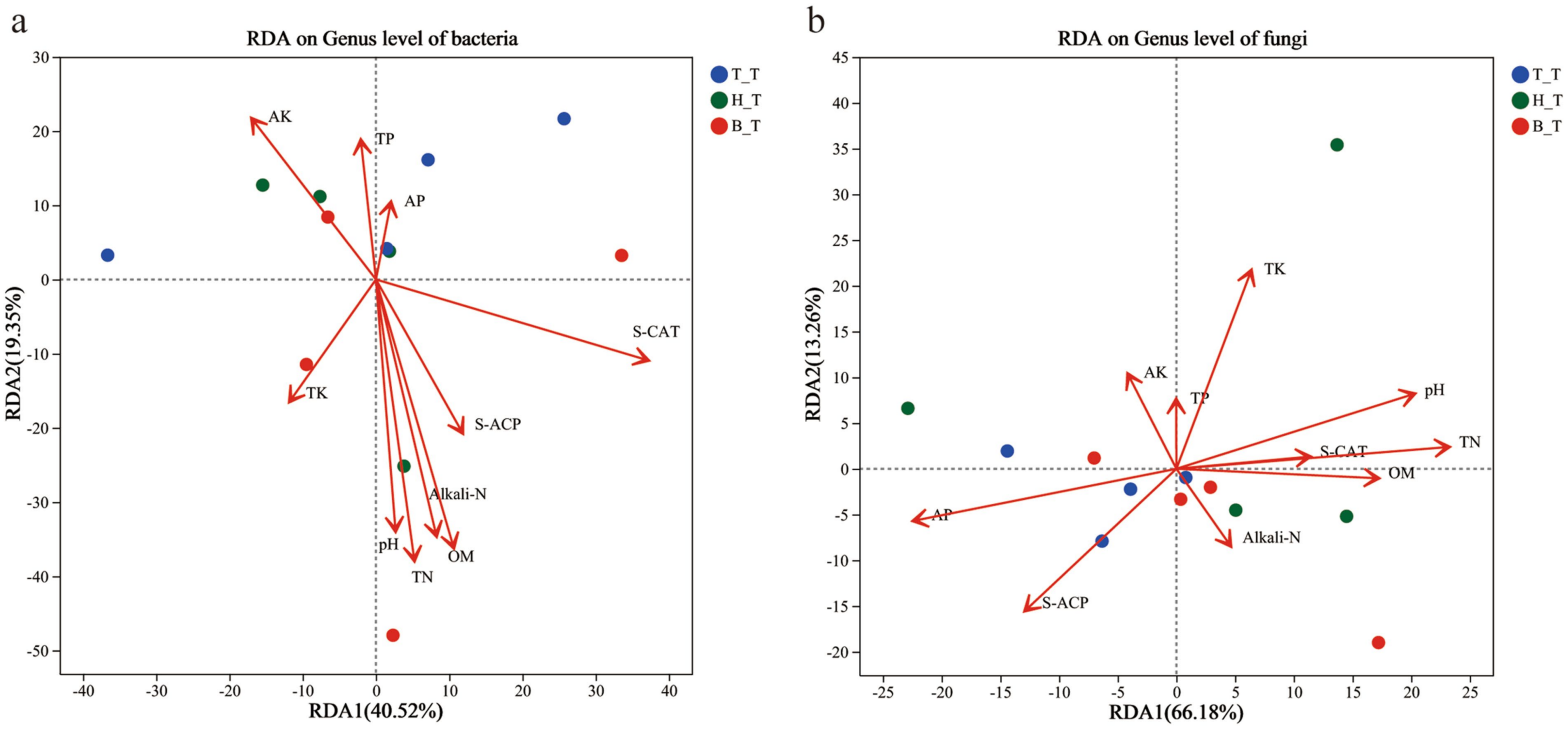
RDA scatter plots of the communities of bacterial **(a)** and fungal **(b)** in inoculated soils with different root exudates added.

RDA was used to analyze the correlation between the community composition of bacterial and fungal genera in the soil with three types root exudates and soil physical & chemical properties and enzyme activity (Figure 6). RDA1 (40.52%) and RDA2 (19.35%) jointly explained 59.87% of the bacterial community variation in the soil, and the top 5 influencing factors are S-CAT, TN, OM, Alkali-N, and pH. For fungi, the cumulative explanatory ratio of RDA1 and RDA2 is 79.44%, and the top 5 influencing factors are TN, AP, pH, TK, and S-ACP. Among which, TN and pH are common influencing factors of bacteria and fungi. S-CAT, OM, and Alkali-N have a significant impact on the distribution of bacterial communities, while AP, TK, and S-ACP affect the distribution of fungal communities. The RDA analysis results showed that the addition of three types of root exudates had a significant impact in the soil physical and chemical properties and enzyme activity on bacterial and fungal microbial communities.

### 3.6 Correlations between metabolites and microorganisms

**Figure 7:**
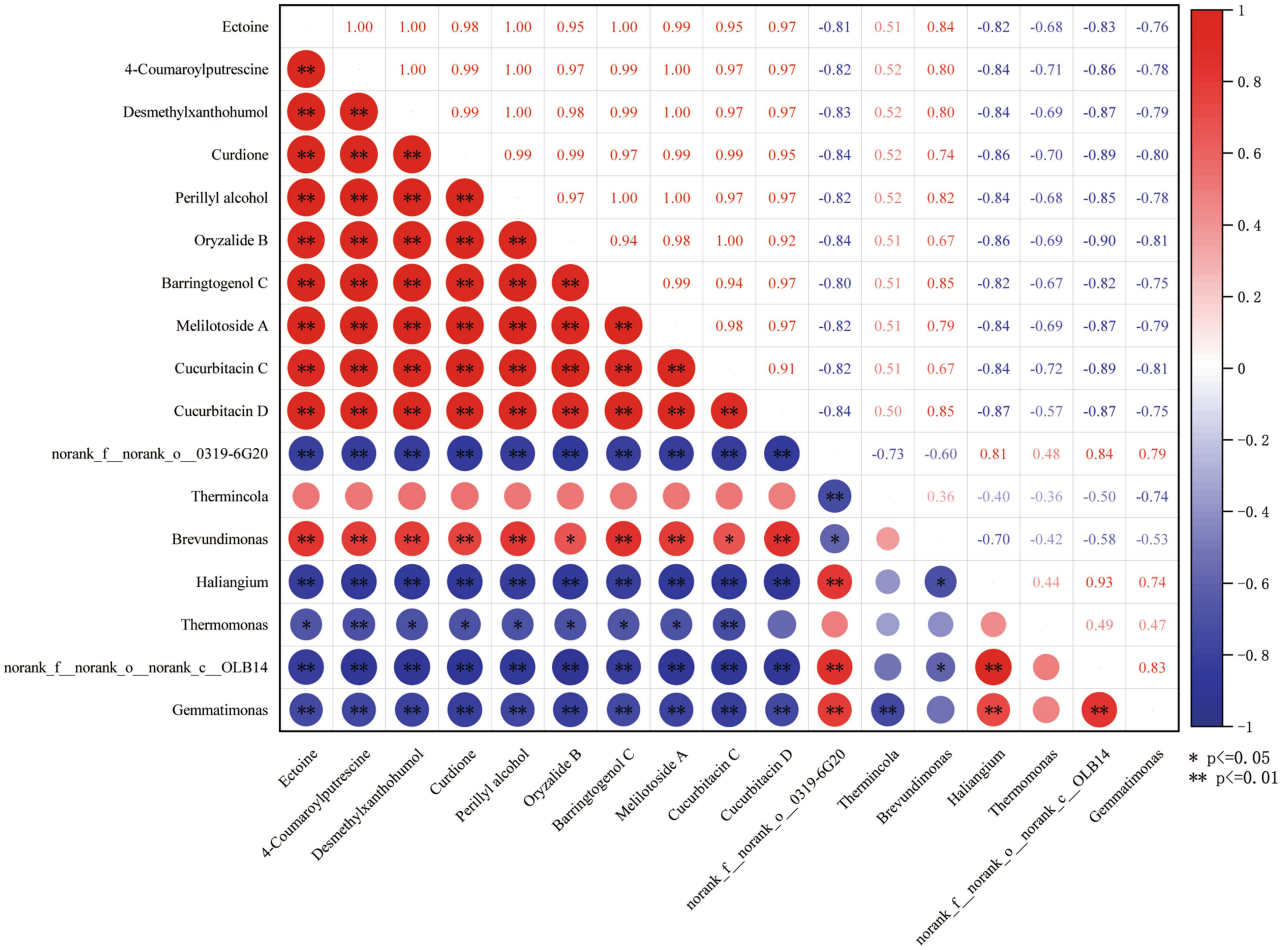
Heat map of metabolite and microbial correlation. * Means that the metabolite is significantly different from the microbe at 0.05 level, ** means that the metabolite is significantly different from the microbe at 0.01 level.

In order to identify the key soil microorganisms affected by root exudates, a correlation analysis was conducted between the 10 metabolites screened and the 7 differentially expressed bacteria screened. As shown in Figure 7, there is a highly significant negative correlation between the 10 metabolites and the 4 differentially expressed bacteria (*norank_f_onorank_o0319-6G20*, *Haliangium*, *norank_f_onorank_cOLB14*, and *Gemmatimonas*); Oryzanide B and Cucurbitacin C, two metabolites, showed a significant positive correlation with *Brevundimonas*; Ectoine, 4-Coumaroylputrescine, Desmethylxanthohumol, Curdione, Perillyl alcohol, Barringtogenol C, Melilotoside A, Cucurbitacin D, eight metabolites, showed a highly significant positive correlation with *Brevundimonas*; Ectoine, Desmethylxanthohumol, Curdione, Perillyl alcohol, Oryzalide B, Barringtogenol C, Melilotoside A, seven metabolites, showed a significant negative correlation with *Thermomonas*; 4-Coumaroylputrecine and Cucurbitacin C metabolites were highly significantly negatively correlated with *Thermomonas*.

Therefore, *norank_f__norank_o__0319-6G20*, *Haliangium*, *norank_f__norank_o__norank_c__OLB14*, *Thermomonas*, *Brevundimonas* and *Gemmatimonas* these six bacteria are key microorganisms that affect soil microecology through root exudates.

## 4 Discussion

### 4.1 Composition difference and functional analysis of root exudates

Plant root exudates are a mixture of various compounds secreted accompanied with the growth of plant, depending on plant species or cultivation methods, developmental stages, plant growth substrates, and stress factors, the quantity and composition of plant root exudates varied accordingly (Hu et al., 2018). In this study, the degree of separation of root exudates from five grafted plants was high and the classification effect was significant (Figure 1), indicating significant differences in metabolites of different root exudates due to different rootstock types or rootstock—scion interaction effect (Romain et al., 2022; Chen et al., 2024).

The most direct response to plant diseases is the alterations of the composition and quantity of root exudates. 56 metabolites with significant differences in relative content that were downregulated in the root exudates of the three experiment objects (H_T, B_T, and T_T) were found in this study. Genreally there are two ways that metabolites enhance plant disease resistance: killing pathogens directly, or shaping favorable soil microecological environments. The study found that exogenous application of Ectoine could improve photosynthetic efficiency and antioxidant activity to enhance the stress resistance of plants (Shang et al., 2017); 4-Coumaroylputrescine possesses the functions of anti-free radical and antifungal activity (Caldas et al., 2019); Desmethylxanthohumol exhibits direct antifungal activity in wheat leaf surface diseases caused by ascomycete fungus Zymoseptoria tritici (Bocquet et al., 2018); Curdione inhibits Phytophthora capsici fungus by disrupting the cell membrane (Wang B. et al., 2019); Perillyl alcohol controls fungal gray mold on tomatoes by inducing the production of jasmonic acid and kaempferol (Zhao et al., 2024); Oryzalide B is an antibacterial diterpene that exhibits resistance to bacterial leaf blight in rice (Kono et al., 2004); Barringtogenol C can increase the abundance and effectiveness of beneficial microorganisms and reduce pathogenic microorganisms in soil (Liu Y. et al., 2024); Melilotoside can be hydrolyzed into coumarin in organisms (Liu et al., 2009), coumarin has antifungal activity (Zaynab et al., 2024); Cucurbitacin has a toxic effect on soil-borne pathogens, thereby enhancing plant resistance (Maja et al., 2022). Therefore, it is inferred that the substances with disease resistance characteristic in the 56 differential metabolites mentioned above are Ectoine, 4-Coumaroylputrescine, Desmethylxanthohumol, Curdione, Perillyl alcohol, Oryzanide B, Barringtogenol C, Melilotoside A, Cucurbitacin C, and Cucurbitacin D. Among them, Melilotoside A is caused by the interaction between pumpkin rootstock and wax gourd scion.

### 4.2 Effects of root exudates on soil properties

Soil pH is a comprehensive reflection of various chemical properties of soil, which affects the absorption and transformation of various nutrients in soil and the activities of soil microorganisms (Fan et al., 2023). Soil pH also affects the growth and colonization of pathogenic fungi. In an acidic environment, pathogenic fungi have a stronger ability to grow and colonize (Wang et al., 2019a; Zhang et al., 2024); The low soil pH stimulates the growth of *R. solanacearum* leads to an increase in the incidence rate of plant diseases (Segura et al., 2021; Mao et al., 2022). This study found that the soil pH was significantly higher when adding H_T and B_T root exudates than when adding T_T root exudates. This may be because root exudates inhibit the growth and colonization of pathogens, regulate the diversity of soil microbial communities (Bi et al., 2022), thus subsequently the occurrence of wilt disease is reduced.

OM can increase increases aeration and water-holding capacity, and enhances plant nutrient cycling and absorption (Wei et al., 2022). Soil with high OM content can enhance microbial activity and functional diversity, thereby increasing inhibition of soil-borne fungal pathogens (Dignam et al., 2019). This study found that the soil OM content was significantly higher with the addition H_T and B_T root exudates. It may be that the addition of H_T and B_T root exudates promotes bacterial growth using soil carbon sources and competes with fungal pathogens for ecological niches (Zhang et al., 2022).

Nitrogen can affect plant resistance and susceptibility to diseases by regulating plant growth and physiological functions, influencing pathogen growth and virulence, and altering the rhizosphere environment (Fagard et al., 2014). Under nitrogen deficient conditions, root exudates reduce soil nitrogen loss by promoting the binding of plants to nitrogen fixing microorganisms and inhibiting the activity of bacteria involved in denitrification (Chai and Schachtman, 2022). Potassium can improve crop tissue structure, thicken thick horn tissue cells, lignify thick-walled cells, increase cellulose content, improve leaf silicification, and effectively hinder pathogen invasion (DeVay et al., 1997; Li et al., 2010). Cotton wilt disease index was highly negatively correlated with soil available potassium concentration, and applying potassium fertilizer can effectively prevent the invasion of fungal pathogens (Zha et al., 2022). In this study, Alkali-N, TN and TK in the soil with H_T and B_T root exudates were significantly higher than that with T_T root exudates, which may promote the formation of soil aggregates, shape a good soil structure, and enhance soil disease inhibition (Li et al., 2021).

In conclusion, the addition of H_T and B_T root exudates can create a soil microenvironment conducive to wilt resistance.

### 4.3 Effects of root exudates on soil microbial community

The *Bacillus* inhibits the infection of FOB by affecting various metabolic pathways, including ABC transporters, amino acid synthesis, and biosynthesis of plant secondary metabolites, significantly suppressing cucumber wilt disease (Liu Z. et al., 2024). In this study, *Bacillus* had the highest relative abundance in the soil with H_T root exudates (Figure 5a), while *Fusarium* had the highest relative abundance in the soil with H_T root exudates (Figure 5c). Compared to the addition of root exudates from T_T, the addition of root exudates from H_T and B_T can reduce the relative abundance of pathogenic bacteria and increase the relative abundance of bacteria which are resistant to FOB.

*Thermincola* is a genus of iron reducing bacteria that participate in the process of organic matter decomposition in soil, degrading organic matter into simpler compounds and releasing nutrients (Zavarzina et al., 2007). *Brevundimonas* has an antagonistic effect against *Fusarium oxysporum* in notoginseng rhizosphere bacteria, making it a beneficial bacterium for resisting root rot (Fan et al., 2016). In this study, the bacteria *Thermincola* and *Brevundimonas* with significant differences in relative abundance (H_T > B_T > T_T) were identified, indicating that the addition of H_T and B_T root exudates is beneficial for increasing the relative abundance of beneficial bacteria that enhance soil nutrient capacity and resist *Fusarium oxysporum*.

In this study, the bacteria with significant differences in relative abundance (H_T < B_T < T_T) were *norank_f__norank_o0319-6G20, Gemmatimonas, Halimium, Thermomonas,* and *norank_f__norank_o__norank_c__OLB14* (Figure 5b). *Norank_f__norank_o__0319-6G20* is a kind of predatory bacterium. In this study, the soil with T_T root exudates had the lowest Alkali-N, TN and TP contents, but the relative abundance of *Norank_f__norank_o__0319-6G20* was the highest in the soil with T_T root exudates. This may be that the soil with T_T root exudates needs to recruit more predatory bacteria to participate in the soil nutrient cycle for nitrogen fixation and phosphorus enrichment (Dai et al., 2023). *Gemmatimonas*, as a potential plant growth antagonist (Obieze et al., 2023), showed the highest relative abundance in the soil with T_T added. The possible reason is more *Gemmatimonas* are required to resist pathogenic bacteria in the soil. *Haliangium* (genus) belongs to the Haliangiaceae (family), which is significantly positively correlated with soil pH and NH_4_^+^-N. In this study, the soil with T_T root exudates had the highest relative abundance of *Haliangium* and the lowest pH, which may indicate that the T_T root exudates need to recruit more *Haliangium* (genus) in the soils to regulate soil pH (Cheng et al., 2022). *Thermomonas* belongs to the denitrifying bacteria (Zhao et al., 2023), *norank_f__norank_o__norank_c__OLB14* (genus) belongs to the Chloroflexi (phylum), which is an important phylum of denitrifying bacteria (Xin et al., 2023), These two bacteria can reduce nitrogen (N) from nitrate (NO_3_^−^) in soil to nitrogen (N_2_) through a series of intermediates (NO_2_^−^, NO, N_2_O), thus resulted in soil nitrogen loss. This may also be the reason why the content of Alkali-N and TN in the soils with T_T root exudates in this study was the lowest.

In conclusion, compared with adding T_T root exudates, the key metabolites present in H_T and B_T root exudates have the potential to directly disrupt the structure of pathogenic fungi and regulate their populations. This process facilitates the release of ecological niches, thereby promoting the recruitment and colonization of beneficial bacteria. Additionally, these metabolites may influence plant physiology, leading to alterations in root exudates that attract beneficial bacteria. Furthermore, they can directly modulate the composition of soil microbial communities, enhancing both the recruitment and maintenance of beneficial bacterial populations through various pathways. Ultimately, this interplay influences soil micro-ecology and contributes to establishing a soil environment that is conducive to resisting wax gourd.

## 5 Conclusion

There were significant differences in root exudates of 5 plants, and 10 key metabolites were found, among which Melilotoside A was caused by the interaction between pumpkin rootstock and wax gourd scion. The addition of root exudates had significant effects on soil pH, organic matter content and nitrogen, indicating that root exudates of resistant plants could shape soil microecology conducive to wilt resistance. In addition, 6 bacteria were found to be key soil microorganisms which influence resistance of grafted wax gourd to wilt disease. This study not only improved our understanding of the resistance mechanism of grafted wax gourd, but also provided a theoretical basis for the research and development of botanical pesticides against wax gourd wilt.

## Data Availability

The datasets presented in this study can be found in online repositories. The names of the repository/repositories and accession number(s) can be found in the article/Supplementary material.

## Footnotes

^1^ http://www.ncbi.nlm.nih.gov/bioproject/1200203

^2^ https://cloud.majorbio.com
