# Supplementary material for: Response of microbial communities and physical and chemical properties in inoculated soils with Fusarium oxysporum Schl. F. sp. Benincasae to different root exudates resistant to fusarium wilt
Source: Front Microbiol. 2025 Jul 17;16:1595426. doi: 10.3389/fmicb.2025.1595426 (PMC12310643; doi:10.3389/fmicb.2025.1595426)
Supplement: Supplementary file 1 [file Table_1.DOCX]

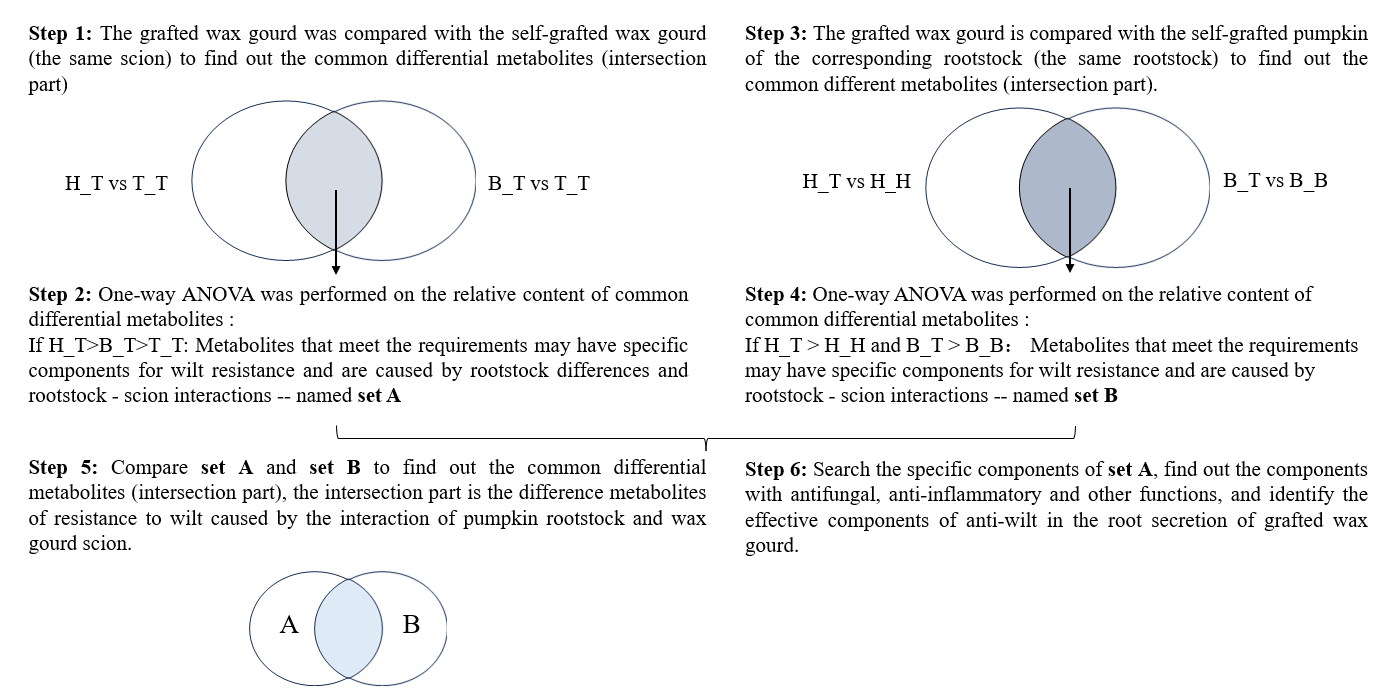


Fig. S1 Analysis of effective components in root exudates resistant to wilt


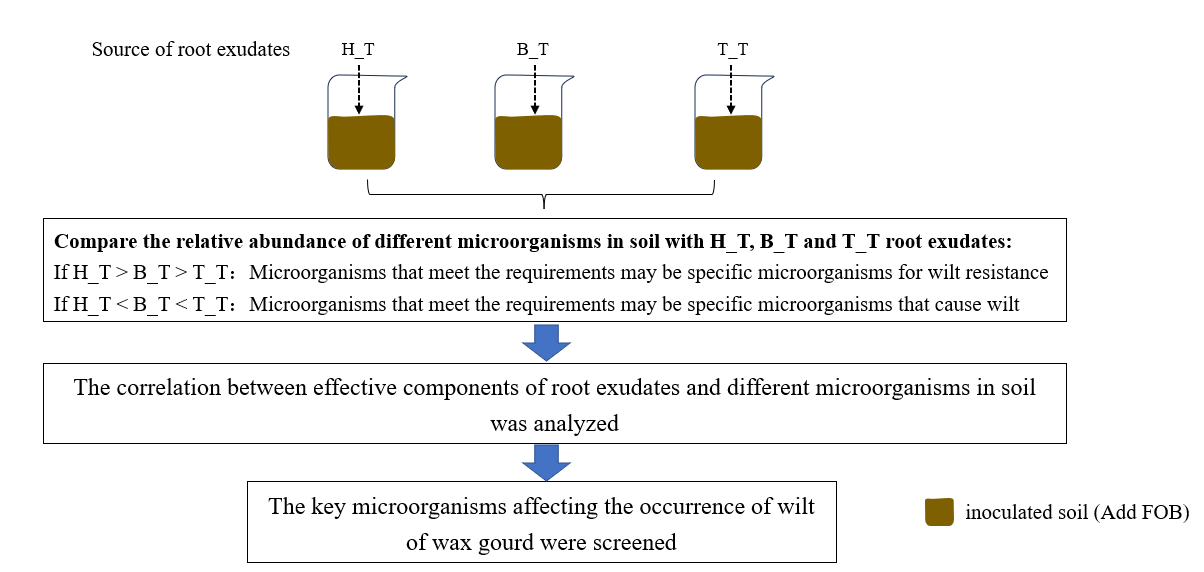


Fig. S2 Analysis of key microorganisms in soil affected by root exudates
